# 1650. Caregiver Perspectives on Educational Materials Used for Paediatric Community-Acquired Pneumonia and Antibiotic Stewardship in the Emergency Department: A Qualitative Study

**DOI:** 10.1093/ofid/ofad500.1484

**Published:** 2023-11-27

**Authors:** Nelson Huang, Lara Murphy, Sujane Kandasamy, Gita Wahi, Jeffrey Pernica

**Affiliations:** University of Toronto, Toronto, Ontario, Canada; University of Toronto, Toronto, Ontario, Canada; McMaster University, Toronto, Ontario, Canada; McMaster University, Toronto, Ontario, Canada; Department of Pediatrics, McMaster University, Hamilton, ON, Canada

## Abstract

**Background:**

The objective of this study was to determine the best modality for educating caregivers on paediatric community-acquired pneumonia (CAP) and antibiotic stewardship within the emergency department (ED) setting. This was to inform the design of educational materials used in a prospective study (PIONEER study, NCT 05114161) testing a novel care pathway for treating non-severe paediatric CAP that aims to reduce unnecessary antibiotic prescriptions.

**Methods:**

This was a phenomenologically-informed qualitative study involving interviews with caregivers of young children in Hamilton, Ontario. Caregivers were presented with four educational materials on appropriate antibiotic prescribing for paediatric pneumonia management. The four materials included: a video of a physician presenting the information, a caregiver discussing her own experiences with antibiotic use for her children, an animated video with audio alongside written information and illustrations, and a brochure. Following this, participants were asked open-ended questions about how they felt about the effectiveness of the materials shown.

**Results:**

Eleven caregivers were interviewed. Most caregivers preferred the combination of the animated video and brochure to the use of either the didactic physician or caregiver video. Participants reported that the stress of the ED environment may hinder their ability to easily process information in the moment. As such, some participants preferred the simpler visuals of the animated video as well as the brochure which they could refer to following their child’s discharge. One caregiver expressed a desire for increasing the diversity of racial identities represented in the educational materials. Although our study focused on paediatric CAP, these findings may have implications in the larger context of caregiver education in the ED.

**Conclusion:**

The findings from this study emphasize the importance of providing clear and concise information to caregivers in the ED environment. Educational aids with simple visuals and concise information which can be referenced following the ED visit are preferred. Additionally, increasing diversity in educational materials can help promote a more inclusive care experience.

**Disclosures:**

**Jeffrey Pernica, MD, MSc, FRCPC, DTMH**, MedImmune: Grant/Research Support|Merck: Grant/Research Support

